# Annotations

**Published:** 1904-02-27

**Authors:** 


### Annotations.

### The Public Health.

The Quarterly Return of the Registrar-General has once more to tell a generally favourable story in relation to the public health. The deaths in England and Wales for the last quarter of 1903 were in the proportion of 16 2 annually per 1,000 persons living, the average death-rate of the ten preceding fourth quarters having been 17·3, which is precisely equal to that for the last quarter of the 76 great towns, each with a population exceeding 50,000, and with an aggregate population of more than 15,000,000. In 103 smaller towns, with populations ranging between 25,000 and 50,000 at the census of 1901, and with an aggregate population estimated at more than 3,500,000 in the middle of 1903, the death-rate for the quarter was 15·5 per 1,000 living, or 1·8 per 1,000 below the rate in the great towns. In the remainder of England and Wales, with a population of more than 14,500,000 of persons, of whom more than 7,000,000 live in small towns, and more than 7,500,000 in rural districts, the death-rate was 15·2. On the other side of the question there was a slight increase in the mortality of children under one year of age, which amounted to 154 per 1,000 of registered births, the average in the ten preceding fourth quarters having been 149. The difference is probably to be explained by the fact that the death-rate from diarrhœa exceeded the average of the ten preceding quarters by 0·08 per 1,000. That of small-pox was identical with the average, and the rates of the other zymotic diseases—measles, whooping-cough, diphtheria, "fever," and scarlet fever—were all below the average. The marriage rate of the third quarter of 1903 was slightly above the average of the last ten third quarters, and the birth-rate of the fourth quarter was slightly below the average. As far as births and deaths are concerned the returns for the last quarter render it possible to complete those for the year, with the result that the death-rate of 1903—15·4 per 1,000—was the lowest on record. Compared with the average rate of the ten years, 1893–1902, it shows a decrease of 2·2 per 1,000. The birth-rate of the year, 28·4 per 1,000 of the population, was also the lowest on record. It was 0·2 per 1,000 below the rate of 1902 and 1·0 per 1,000 below the average of the ten preceding years.

### Premature Burial.

It is not necessary to admit the accuracy of all the statements advanced in reference to the occurrence of premature burial in order to sympathise with many of the proposals advanced by the Association which exists for the prevention of so terrible a disaster. There can be no question that the conditions which govern the present arrangements for the burial of dead bodies are in many respects highly unsatisfactory. It is said that as many as 10,000 bodies are buried each year without any certificate of death. Further, the official certificate which the law compels the medical practitioner to furnish does not provide that he shall certify the fact of death on the basis of his own observation. It is sufficient that he has been in professional attendance on a patient and that he has been "informed" that death has actually taken place. He is not called upon to examine the body much less to apply any of the scientific tests by which the proof of death may be established. The law indeed regards the duty of death certification as so slight an affair that whilst it compels the medical practitioner to undertake its formal discharge it does not regard the proceeding as worthy of even a nominal financial recognition. In all this it is obvious there is great opportunity for error and even for crime, and the profession will therefore be disposed to sympathise with the efforts of the Association for the Prevention of Premature Burial to carry through Parliament a bill enacting that no certificate of death shall be given without a personal examination and inspection of the body. A second clause in the draft bill provides for the establishment of public waiting mortuaries, where bodies are to be kept until the fact of death is placed beyond dispute by the signs of commencing decomposition, and this too has a wide claim on sanitary grounds and apart from the special object of the Association. Whether well founded or not it is an undoubted fact that many people suffer much unhappiness under the fear that they may possibly be buried alive. Even if the risk is as slight as most authorities contend, there is no reason why it should not be completely removed, and if in order to secure this improved regulations regarding the granting of death certificates and sanitation are essential the Association for the Prevention of Premature Burial may well be regarded as worthy of support.

### Drugs and the War.

The commercial disturbances produced by the war in the Far East are not likely seriously to affect the drug market except in regard to two substances, namely, camphor and menthol, for the supply of which Japan holds a practical monopoly. The supply of linseed from Russia may, the *Pharmaceutical Journal* thinks, be somewhat disturbed, but for the present both this and menthol are little if at all affected. Camphor, on the other hand, shows a strong upward tendency, and this is the more unfortunate seeing that for many months the supplies have been scanty and the price correspondingly high. The Japanese Government now controls the camphor industry in Formosa, and the drug is three to four times the price at which it was quoted ten years ago. The war may be expected to drive the price still higher, unless an announcement, which comes from the United States, shall prove to be capable of neutralising this tendency. This announcement is to the effect that experiments show that it is quite possible to prepare camphor by a synthetic process in the chemical laboratory, and there seems every reason to believe that this can be done in such a way as to ensure a practical commercial success. The chemical details are somewhat elaborate, and afford a good example of scientific patience and determination directed to a particular end. It is much to be hoped that the anticipations suggested by this new departure will be realised. Camphor, of course, has many medicinal and commercial applications, and the consumer has already been penalised by the arrangements which leave its production as a monopoly in the hands of a commercial syndicate. If this is made to feel the pressure of an effective competition a rapid fall in prices may be expected.

